# Unusual hybrid repair of a thoracoabdominal and mesenteric aneurysm with aberrant right hepatic artery

**DOI:** 10.1016/j.jvscit.2021.05.016

**Published:** 2021-06-04

**Authors:** Milán Vecsey-Nagy, Zoltán Szeberin, Csaba Csobay-Novák

**Affiliations:** a Heart and Vascular Center, Semmelweis University, Budapest, Hungary; b Department of Vascular and Endovascular Surgery, Semmelweis University, Budapest, Hungary; c Semmelweis Aortic Center, Semmelweis University, Budapest, Hungary; d Department of Interventional Radiology, Semmelweis University, Budapest, Hungary

**Keywords:** Thoracoabdominal aortic aneurysm, Visceral aneurysm, Hybrid approach, Visceral debranching, Stent graft

## Abstract

Hybrid approaches for the treatment of thoracoabdominal aortic aneurysms that combine visceral debranching and endovascular repair are feasible alternatives to open surgery for certain high-risk patients. A 70-year-old man was admitted with a rapidly expanding thoracoabdominal aneurysm involving the superior mesenteric artery, associated with an aberrant hepatic artery. An iliovisceral debranching was performed, followed by the endovascular repair of the thoracoabdominal aorta with a standard thoracic device. The ostial aneurysm was excluded by retrograde implantation of a covered stent from the superior mesenteric artery. Such approach can be considered as a viable alternative in the management of complex thoracoabdominal aneurysms.

The conventional surgical repair of a thoracoabdominal aortic aneurysm (TAAA) is a high-risk operation associated with substantial morbidity and mortality. The majority of patients presenting with a TAAA are elderly with significant comorbidities and generally considered to be suboptimal candidates for open surgery. The widespread availability of thoracic endovascular devices allowed for the emergence of hybrid procedures, combining surgical debranching of visceral arteries and subsequent endovascular aneurysm repair. The first reported hybrid management of a TAAA was published in 1999, and although the emergence of a wide array of fenestrated and branched endografts offers the option of total endovascular repair for a specific subgroup of patients, this approach has remained a reasonable alternative to high-risk patients who are neither candidates for total endovascular repair nor open surgery.^1^^,^^2^ Patients with concomitant involvement of visceral vessels, however, pose a particular challenge for endovascular repair and require further considerations. The patient provided informed consent for the publication of his case details and images.

## Case presentation

**Fig 1:**
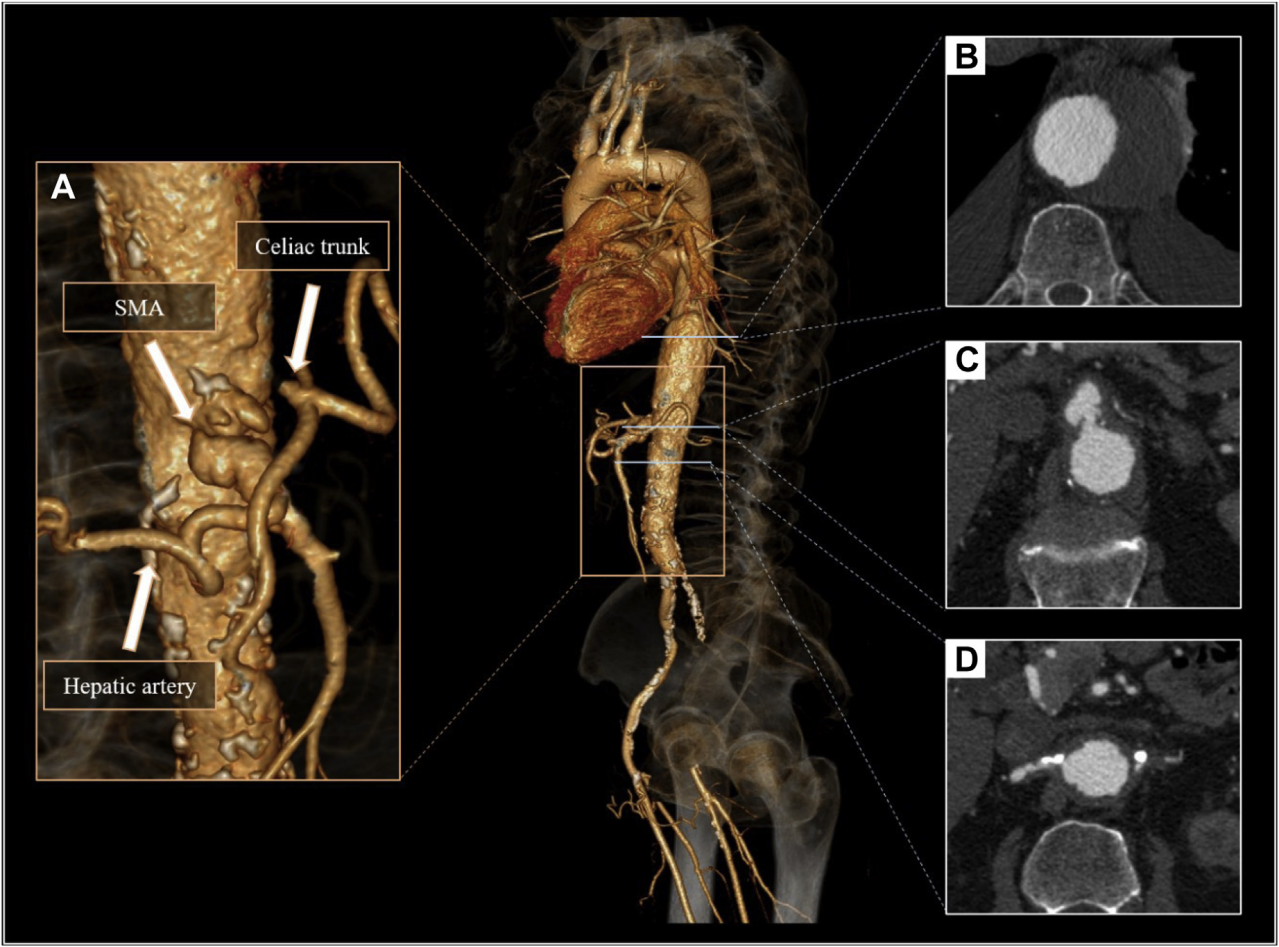
Preprocedural computed tomography (CT) angiography. **A,** Volume rendered image of the aorta displays the pseudoaneurysm of the superior mesenteric artery (*SMA*), aberrant right hepatic artery, and the occlusion of the celiac trunk. **B,** Thoracoabdominal aneurysm with a maximal diameter of 70 mm. **C,** Ostial pseudoaneurysm of the superior mesenteric artery. **D,** Occlusion at the origin of the left renal artery.

**Fig 2:**
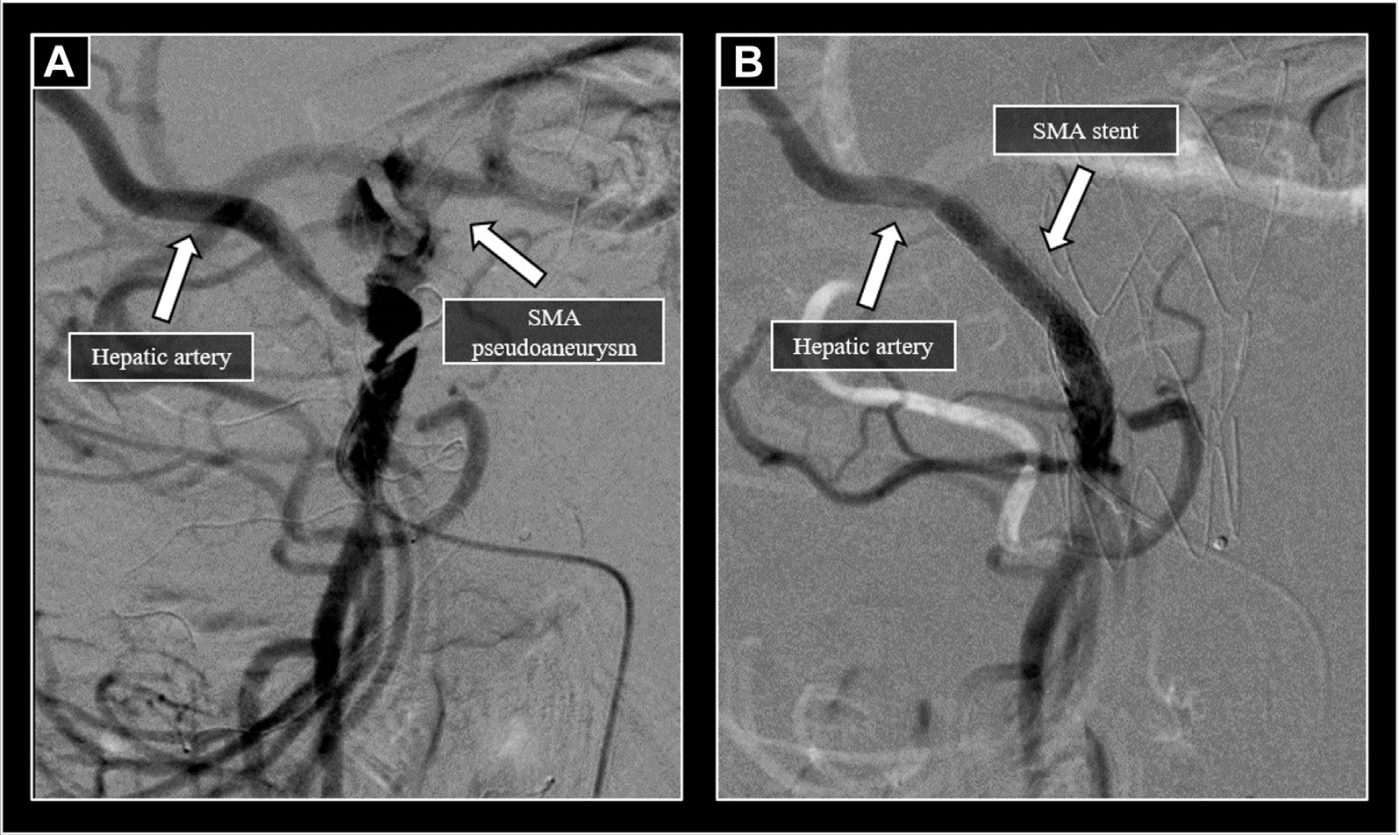
Selective angiography of the superior mesenteric artery (*SMA*) after the deployment of the thoracoabdominal stent graft. **A,** Active filling of the pseudoaneurysm at the origin of the SMA was confirmed. **B,** After the implant of a covered stent, the pseudoaneurysm was excluded from circulation and adequate filling of the hepatic branch was maintained.

A 70-year-old male patient was admitted to our center for a routine computed tomography (CT) scan after transurethral resection of a bladder tumor. He had a medical history of hypertension, ischemic heart disease, chronic kidney disease, and peripheral artery disease. Besides the previously documented occlusion of the left common iliac artery, an asymptomatic TAAA was discovered with a diameter of 60 mm. The patient initially declined the recommended operation. CT angiography described a substantial progression a year later: an increase of the aortic diameter (70 mm), and an ostial aneurysm of the superior mesenteric artery (SMA) was noted with the chronic occlusion of the celiac trunk and the left renal artery (Fig 1). Aortic team recommended a hybrid repair consisting of iliovisceral debranching followed by endovascular aortic repair of the visceral aortic segment with a standard thoracic device and subsequent covered stent placement into the SMA, leading to the aberrant right hepatic artery. The patient agreed to undergo the procedure, and written informed consent was obtained. Visceral debranching was achieved via midline laparotomy. Using prosthetic conduits, retrograde bypass was constructed using a 12 mm × 6 mm bifurcated Dacron graft (Gelsoft; Vascutek/Terumo, Scotland, UK) connecting the right common iliac artery with the right renal artery and the SMA. Initial proximal ligation of the right renal artery was followed by an end-to-end anastomosis between the graft and the right renal artery. Thereafter, the SMA was reconstructed in an end-to-side fashion with the other limb of the conduit. A 36 mm × 200 mm Valiant device (Medtronic Vascular, Santa Rosa, Calif) was subsequently deployed to exclude the TAAA, covering the visceral segment of the aorta. Finally, a 6 mm × 38 mm covered stent (BeGraft; Bentley Innomed GmbH, Hechingen, Germany) was deployed in the SMA ending in the right hepatic artery via the prosthesis. Completion angiography showed complete exclusion of the aneurysm sac (Fig 2).

Postoperative therapy was supplemented with clopidogrel, which was discontinued after 90 days. Furthermore, the patient received pharmacologic thromboprophylaxis (enoxaparin) until his discharge. Given that early postoperative assessment of the patient did not confirm neurologic deficit, cerebrospinal fluid drainage was not administered.

**Fig 3:**
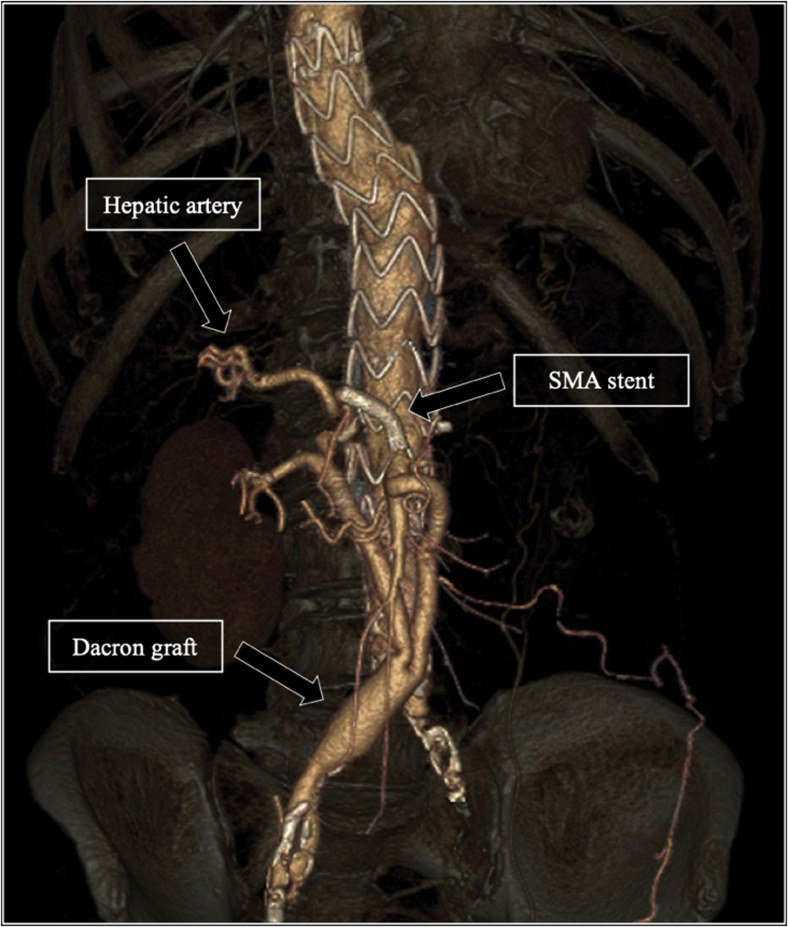
Follow-up computed tomography (CT) angiography of the aorta 12 months after the procedure showing patency of the Dacron graft and the superior mesenteric artery (*SMA*) covered stent.

After an uneventful postoperative period, the patient remained in the intensive care unit for 4 days and was discharged on the 11th day. Although the previously documented mild chronic kidney disease was manifested on the baseline renal function of the patient (glomerular filtration rate: 38.2 mL/min), it did not significantly decrease further on the following postoperative days (glomerular filtration rate: 29.7 mL/min at discharge). Follow-up CT angiography at 2 and 12 months confirmed patent grafts with no sign of endoleak (Fig 3). The patient was asymptomatic 24 months after the procedure.

## Discussion

Despite continuous evolution of surgical techniques and strategies, open surgical repair of a TAAA is still a high-risk operation, associated with substantial morbidity and mortality rates. The in-hospital mortality rate of open thoracoabdominal aortic aneurysm repair is reported to be 11%.^3^ The mortality risk, however, further increases with patient age and the number of comorbidities and is also associated with the volume of cases performed in a center. Spinal cord ischemia and renal failure are reported to be the most common complicating factors accounting for the formidable nature of surgical undertaking.^4^

Endovascular repair of thoracoabdominal aneurysms using fenestrated and branched devices is becoming increasingly popular, as the mortality rate is lower than in open surgery, being as low as 4% even in patients with redo aortic operations, as published recently.^5^ In our case, however, fenestrated or branched repair (such as the octopus technique) was not possible as there was no distal landing zone for an SMA bridging element proximal to the offtake of the aberrant right hepatic artery. A further consideration of treatment selection was that the left common iliac artery was chronically occluded; thus bilateral access route was not available for complex endovascular repair.

The combined endovascular and surgical approach to thoracoabdominal aortic pathology has been introduced as a less invasive alternative that has the advantage of avoiding thoracotomy, single-lung ventilation, aortic cross-clamping, and prolonged end-organ ischemia.^6^ It is especially useful in cases of urgent repair, with the devices being readily available compared with the average production time of 6 weeks for custom-made devices. The hybrid strategy remains a viable alternative for certain high-risk patients who are neither candidates for total endovascular repair nor open surgery.^7^ Hughes et al^8^ reported outstanding outcomes of 58 patients who underwent hybrid repair. A perioperative mortality rate of 9% was documented and the rate of permanent paraplegia was 4%. Over a mean follow-up of 26 months, visceral graft patency was 95.3%, and all thromboses were clinically silent. Quinones-Baldrich et al^9^ published their overall experience investigating a mixed cohort of high-risk patients undergoing hybrid procedures. They documented substantial advantages of hybrid repair, with regard to morbidity, mortality, and need for subsequent intervention. The overall survival rate was 76% at 2 years. Additional involvement of visceral arteries, however, further complicates selection of the optimal repair strategy.

This particular case posed a significant challenge regarding its management, given the combination of a rapidly progressing thoracoabdominal aneurysm, the ostial aneurysm of the SMA with an aberrant right hepatic artery, and the occlusion of both the celiac trunk and the left renal artery. Considering the presence of severe visceral artery disease at multiple locations, organ ischemia associated with surgical repair represented a substantial risk. Intraoperative ischemia to visceral branches may lead to renal failure and coagulopathy, both associated with increased mortality.^10^ Because clamping of the aorta was avoided, ischemia time to each of the visceral arteries was limited.

Intraoperative angiography confirmed retrograde filling of the aneurysm sac originating from the SMA. Performing a bypass on an SMA with a large ostial lesion that is not isolated proximally from the aortic aneurysm carries substantial inherent risk, as it could lead to persistent backflow into the aneurysm sac. Eventually, an unconventional method for the backdoor exclusion of the aneurysm was selected, after visceral debranching and endovascular aortic repair.

In summary, this report demonstrates the case of a rapidly expanding thoracoabdominal and SMA aneurysm, successfully treated by an unusual hybrid repair. Further evidence supporting our experience may establish this approach as a viable alternative in the management of thoracoabdominal aneurysms and concomitant visceral aneurysms.
